# 24 h Holter ECG Monitoring of Patients with Rheumatoid Arthritis—A Potential Role for a Precise Evaluation of QT Interval Duration and Associated Arrhythmic Complications

**DOI:** 10.3390/diagnostics12030638

**Published:** 2022-03-05

**Authors:** Elena E. Saramet, Robert D. Negru, Andra Oancea, Maria Magdalena Leon Constantin, Codrina Ancuta

**Affiliations:** Department of Internal Medicine, Grigore T. Popa University of Medicine and Pharmacy, 700115 Iasi, Romania; smaranda.saramet@gmail.com (E.E.S.); andra.radulescu@yahoo.com (A.O.); leon_mariamagdalena@yahoo.com (M.M.L.C.); codrina_ancuta@yahoo.com (C.A.)

**Keywords:** rheumatoid arthritis, Holter ECG, QTc interval, QTc dynamics

## Abstract

Background: Patients with rheumatoid arthritis (RA) have increased systemic inflammatory burden associated with elevated cardiovascular mortality. Prolonged ventricular repolarisation evaluated by QT interval duration is a risk factor for cardiovascular and total mortality. In RA, mortality risk is correlated with dynamics and cumulative incidence of QTc prolongation rather than QTc value. The aim is to evaluate if QT parameters evaluated with 24 h Holter ECG are a better option to complete the cardiovascular profile of RA patients than parameters from short ECG recordings. Materials and methods: A total of 58 patients (22 males, 36 females) with RA were submitted to short ECG recordings at admission and to 24 h Holter ECG. QT interval parameters and ventricular ectopy generated from both types of recordings were analyzed. Results: QTc interval values obtained from Holter ECG were significantly higher than the values from short term ECG and were correlated with severity of inflammatory process. The number of QRS complexes with QTc > 450 ms recorded during 24 h Holter was strongly correlated with the number of ventricular events and severity of the inflammatory process. Conclusions: In patients with RA, the Holter ECG recordings could realize a more precise evaluation of the extent and dynamics of QTc interval duration and of ventricular ectopic events with potential risk of sudden death.

## 1. Introduction

As a chronic inflammatory disease, rheumatoid arthritis (RA) is defined by joint swelling and tenderness and destruction of synovial joints, which are associated with severe disability and premature mortality [1]. The death risk of the patients with RA is higher when compared with similar members of the general population, the excess of mortality being caused mainly by cardiovascular diseases [2,3]. Patients with RA experience sudden cardiac death (SCD) risk at a rate that is two times higher when compared with normal subjects (6.7% vs. 3.8%) [4]. In patients with systemic autoimmune diseases, the prevalence of QT interval prolongation can be up to 30% [5]. In RA, QT interval prolongation and arrhythmic risk are increased by chronic systemic inflammation responsible for an accelerated atherosclerotic process, followed by ischemic heart disease, but there is increasing clinical and experimental evidence that inflammation is associated with abnormal ventricular repolarization and cardiovascular autonomic system dysfunction (increased sympathetic outflow with stimulation of β1 adrenergic receptors on myocytes), which contribute to an increased rate of ventricular arrhythmias associated with the increased rate of sudden cardiac death (SCD) observed in patients with RA [6,7,8,9]. The direct effect of inflammation on ventricular repolarization and QT duration is mediated by the action of inflammatory cytokines on calcium and potassium channels in myocytes with alteration of action potential duration (APD) [5]. Studies confirmed that TNFα, IL-6 and IL-1 increase the APD duration [6]. There are even recent studies that suggest that the evaluation of components of QT interval are a better option to evaluate the risk for SCD [10]. The heart rate-corrected value of the QT interval duration (QTc) is still widely used as a measure of ventricular repolarization; a QTc prolonged value reflects an abnormal repolarization and an increased arrhythmic potential associated with an increased risk of SCD [11,12]. In clinical practice, standard electrocardiographic recordings are usually used for heart disease screening and to evaluate the duration of QTc interval; however, in many cases, they fail to detect any early cardiac abnormalities [13]. Our study aimed to evaluate if the 24 h Holter electrocardiographic monitoring in patients with RA could provide additional useful parameters that could contribute to a better understanding of the cardiovascular profile of these patients.

## 2. Materials and Methods

Our study included 58 patients with rheumatoid arthritis admitted in the II-nd Rheumatology Clinic Clinical Rehabilitation Hospital Iasi between January 2019 and November 2021. The study included patients diagnosed with RA according to the ACR/EULAR 2010 rheumatoid arthritis classification criteria, confirmed by clinical examination and laboratory tests. The patients with associated pathology or treatments that can interfere with QT interval duration or HRV values, such as ischemic heart disease, arterial hypertension, cardiac failure, chronic pulmonary diseases, diabetes, atrial fibrillation, bundle branch block, presence of the treatment with beta blockers or antiarrhythmic drugs, were excluded from our analysis. No treatment with TNF-α antagonists or IL-6 inhibitors were administered for at least 30 days before the admission nor during the electrocardiographic examinations. The study protocol was approved by the Ethics committee of the hospital and all patients signed an informed consent when included in the study. Following the standard procedure of the hospital, for all the patients included in the study, a short ECG recording (10 s) was realized using a BeneHeart12 (Mindray) electrocardiograph. The equipment also realized the automatic calculation of QT and QTc intervals routinely used for cardiovascular evaluation of the patients. All the recordings were analyzed by one of the authors for ST elevation or depression and for the presence of ventricular ectopic events. During the admission, all the patients were submitted to a 24 h Holter ECG recording using DM Software—USA software and recorders (Cardioscan 12 Premier and 300-3A recordings with 10 electrodes/12 leads). No nicotine or coffee consumption was allowed during the 24-h monitoring. One author had revised all the Holter recordings before automatic analysis was performed by the software. The Holter software automatically calculates the QTc interval duration for all the recorded sinusal beats; in our study, we used the following parameters generated by the software: the mean value of QTc interval obtained for all the sinusal beats recorded during Holter ECG monitoring and maximal value of QTc (max QTc) and QT (max QT) intervals recorded during Holter monitoring. For each patient, the software calculates the percentage of sinusal beats (reported to the total number of sinusal beats recorded at the patient) having the absolute QTc interval duration included in 10 predefined ranges (from <350 ms to >510 ms). In our study, the number of female patients was dominant, and we set the reference QTc value at 450 ms for interval prolongation. For each patient, the total number of sinusal beats recorded during Holter monitoring with QTc > 450 ms was calculated. For each patient, total time (minutes) of ST interval changes (>1 mm elevation/depression) recorded in at least one lead during the 24 h Holter recordings was calculated by the software. The number of ventricular events (ventricular ectopic beats and ventricular tachycardia episodes) was recorded separately. For the statistical analysis, we used OpenSTAT and PSPP software. The data are presented as median and range or means ± standard deviation (SD). Normal distribution of the parameters was tested using the Shapiro–Wilks test. The t-Student and one-way ANOVA F parametric tests were used for all the parameters that had a normal distribution. The Mann–Whitney U test was used for non-normal distribution. Correlations were evaluated using Spearman or Pearson coefficients, as appropriate. Statistical significance was set at a *p* value of 0.05 or less.

## 3. Results

A total of 58 patients (35 females and 22 males) with rheumatoid arthritis were included in our study. The mean age of the patients was 57.97 ± 8.59 years. According to the Sharp radiological scores of RA, 2 patients (4%) were in stage I, 24 (42%) in stage II, 28 (48%) in stage III and 4 (6%) in stage IV. The biochemical and haematological characteristics of the patients included in the study are presented in Table 1.

On standard ECG recordings, none of the analyzed patients had a QTc interval duration longer than 450 ms. The mean value of the QTc interval obtained using this type of recording was 401.55 ± 23.70 ms. There is no statistical difference (*p* = 0.104) between the value of the QTc interval duration recorded in female patients when compared to males. In one female patient, ventricular bigeminy was present and no pathological changes of ST interval were present on short term ECG recordings. In addition, we weren’t able to find a significant statistical difference regarding the value of the QTc interval between patients with normal (0–5.0 mg/L) and elevated levels of C-reactive protein (>0.5 mg/L).

Using 24 h Holter ECG recordings, the median of the number of sinusal beats analyzed for each patient was 92,926 (range: 52,332–135,833). During Holter ECG monitoring, all the patients presented sinusal beats with QTc > 450 ms; in 32 patients, QTc values higher than 510 ms were recorded. For each patient, we calculated the number of sinusal beats with QTc > 450 ms recorded during the Holter monitoring; the median was 34,059 beats (range 245–126,979). Mean value of the QTc interval generated by the analysis of the sinusal beats recorded on Holter ECG recordings was 445 ± 19.94 ms, significantly higher (*p* < 0.05) than the mean QTc value recorded when short ECG recordings were used. A comparison between Holter electrocardiographic parameters recorded in male and female patients included in the study is presented in Table 2.

The ventricular ectopic contractions and ST segment changes identified on Holter ECG are presented in Table 3.

Only the number of sinusal QRS with QTc > 450 ms recorded during 24 h monitoring was significantly correlated with the number of premature ventricular contractions (PVC) recorded during monitoring. We compared the electrocardiographic parameters of the patients according to the C-reactive protein (CRP) level. The results are presented in Table 4.

We analyzed if the electrocardiographic parameters correlated with the inflammatory status of the patients expressed by the level of the CRP and erythrocyte sedimentation rate (ESR). The results are presented in Table 5. No significant correlations were identified for ESR.

## 4. Discussion

In rheumatoid arthritis, chronic inflammation is associated with an accelerated development of the atherosclerotic plaque; however, there is evidence that inflammation can be associated with electrophysiological abnormalities, which can directly generate arrhythmic events associated with death risk [6,14]. QT interval prolongation is an important predictor for arrhythmic events associated with SCD [15]. The prognostic value of the QTc interval was confirmed for individual clinical situations [16,17,18,19], including RA [20]. In clinical practice, the duration of the QT interval is evaluated using standard short electrocardiographic recordings; clinical studies have shown that, when short recordings were used, the value of the QTc interval in patients with RA was significantly longer when compared with controls [9,21,22], even when there was no significant difference concerning the prevalence of QTc prolongation between patients with RA and controls [12]. In our study, when short electrocardiographic recordings were used, only four patients had values of QTc > 440 ms and no QTc > 450 ms was recorded. Our results were similar to Goulenok et al. [13], who used short ECG recordings, in a study including 106 patients with RA and did not find any QTc > 450 ms, and were also similar to those of Erre et al. [12] and Aladag et al. [23], who could not confirm an association between RA and prolonged QTc. In our study, there were not significant differences between the QTc interval values recorded in male and female patients. Our results are not concordant with the data obtained by Panoulas et al. [20], who, in a study involving more than 350 patients with RA using short recordings, found a percentage of 6.7% patients having a QTc > 450 ms. Our results were also similar to those of Lazzerini et al. [24], who documented a significantly higher value of QTc in females when compared with males, when 15 min electrocardiographic recordings were used to extract QTc values. These differences can be explained by the exclusion criteria used in our study and in the one realized by Erre et al., which was more restrictive when compared to the ones applied in the studies of Panoulas and Lazzerini. Similar to Erre et al. [12], we were not able to confirm any correlations between QTc value generated using short ECG recordings and RA activity expressed by CRP or ESR levels, as identified in other studies using the same type of recordings [25]. 

There is increasing evidence suggesting that various parameters related to time variation and to dynamics of the QT interval extracted from Holter ECG recordings may have a more important predictive power compared to the absolute value of the QTc interval [26,27], a fact that confirms the utility of Holter ECG recordings in the evaluation of QT intervals. Erre et al. confirmed that an increased QT interval dispersion (QTd) was independently associated with RA and that patients with RA had a significantly higher QTd value and prevalence when compared with controls [12]. Chauhan et al. [25] found an increased cumulative incidence of QTc interval prolongation in patients with RA when compared with controls and a slight correlation of this parameter with all-cause mortality. The importance of variation of the QTc interval duration was confirmed also by Panoulas et al. [20], who found that, in patients with RA, an absolute increase of 50 ms in QTc interval duration was associated with a twofold increase of all-cause mortality. A recent cohort study suggests the potential utility of monitoring the QTc changes over a longer period of time (7.6 ± 2.6 years) to predict increased risk for cardiovascular events [28].

When Holter recordings were used, the mean value of the QTc intervals analyzed was significantly higher than the mean value generated by the short recordings (445 ± 19.94 ms vs. 401.55 ± 23.70 ms, *p* = 0.0001), but we did not identify any significant difference (*p* = 0.068) when compared with the reference value of 450 ms. The Holter results showed that all patients had sinusal beats with QTc > 450 ms, with a median value of 34,059 beats (range 245–126,979). These results confirmed the dynamic profile of the QT interval, a characteristic that short electrocardiographic recordings used in clinical practice fail to evaluate. Both mean QTc value and the number of sinusal complexes with QTc > 450 ms were positively correlated with CRP levels, but we could not confirm a similar correlation with ESR levels. Moreover, the values of these two electrocardiographic parameters are significantly higher in patients with CRP > 5 mg/dL, when compared with patients with normal CRP levels. These results are similar to other studies [9,20,24] confirming the link between inflammatory process and QTc interval prolongation. Only the number of the sinusal QRS with QTc > 450 ms recorded during 24 h monitoring was significantly correlated with the number of premature ventricular contractions (PVC), a correlation confirmed also in patients with systemic sclerosis [29]. In our study, the percentage of RA patients (54%) with ventricular arrhythmic events was similar to the results of previous studies that used 24 h Holter monitoring, studies which were not able to identify any significant differences between patients with RA and controls with respect to these events [30,31]. 

The limitations of our study are related to the relatively small number of patients included in the study and to the possibility that QTc parameters generated by the used Holter ECG software are not available with other systems. These limitations suggest that the clinical utility of our results may be restricted to selected patients. 

## 5. Conclusions

In patients with RA, the short ECG recordings used for the evaluation of the QTc interval may underestimate the presence of prolonged QTc interval. Parameters extracted from 24 h Holter ECG recordings—such as the number of sinusal QRS with QTc > 450 ms—correlated with the inflammatory status of the patient, could realize a more precise evaluation of the time variations of QTc interval duration and could contribute to a more precise cardiovascular profile of the patient with RA.

## Figures and Tables

**Table 1:** Biochemical and haematological profiles of the patients included in the study.

Parameter (SI Units)	Value (Mean ± SD)
Glucose (mg/dL)	96.54 ± 21.61
Uric Acid (mg/dL)	3.90 ± 1.20
Blood Urea Nitrogen (mg/dL)	36.80 ± 12.39
ALAT (U/L)	20.34 ± 8.16
ASAT (U/L)	20.72 ± 4.39
Gamma Glutamyl-transpeptidase (U/L)	28.75 ± 18.55
Total Cholesterol (mg/dL)	187.25 ± 36.98
Triglycerides (mg/dL)	113.59 ± 46.77
Creatinine (mg/dL)	0.81 ± 0.14
Alkaline Phosphatase (U/L)	162.65 ± 68.65
C-reactive protein (mg/L)	5.24 ± 5.67
VSH	35.10 ± 24.88
WBC (×10^3^/mm^3^)	7.33 ± 2.10
RBC (×10^6^/mm^3^)	4.28 ± 0.50
Hemoglobin (g/dL)	12.60 ± 1.17
Platelets (×10^3^/mm^3^)	304.69 ± 79.21
Lymphocites (×10^3^/mm^3^)	2.05 ± 0.91
Neutrophils (×10^3^/mm^3^)	4.55 ± 1.70
Monocytes (×10^3^/mm^3^)	0.44 ± 0.17
Eosinophils (×10^3^/mm^3^)	0.19 ± 0.14
Basophils (×10^3^/mm^3^)	0.09 ± 0.04

**Table 2:** Holter electrocardiographic parameters male vs. female patients.

ECG Parameter	Males (no = 22, 38%)	Females (no = 36, 62%)	*p*
Sinusal QRS complex analysed (no)	94 657	97 717	NS
Sinusal QRS with QTc > 450 ms (no)	36 817	50 113	NS
QTc (ms)	442.55 ± 24.52	446.72 ± 16.73	NS
Max QT (ms)	466	494	NS
Max QTc (ms)	504	540	0.0024

**Table 3:** Ventricular ectopic and ST segment events recorded on Holter ECG.

ECG Holter Event	
PVC n/24 h (median-range)	99 (0–18,666)
Patients with PVC n (%)	54 (93.1%)
Patients with PVC > 1000/24 h n (%)	4 (6.9%)
Patients with TV non sustained n (%)	0 (0.0%)
Pacients with ventricular bigeminy or trigeminy n (%)	2 (3.44%)
Patients with ST elevation/depression > 1.0 mm (min)	48 (82.75%)

**Table 4:** Electrocardiographic parameters generated by 24 h Holter ECG compared according to CRP level of the patients.

ECG Parameter	Patients with CRP > 5 mg/dL (n = 20, 34%)	Patients with CRP < 5 mg/dL (n = 38, 66%)	*p*
Premature ventricular beats (PVC)	361	1139	0.03
Sinusal QRS with QTc > 450 ms (no)	67,669	30,419	0.0005
QTc (ms)	453.7 ± 16.88	440.63 ± 20.14	0.013
Max QT (ms)	496.90 ± 79.61	477.32 ± 50.35	NS
Max QTc (ms)	542.3 ± 69.34	518.79 ± 47.94	NS

**Table 5:** Correlations between electrocardiographic parameters and CRP blood levels.

ECG Parameter	CRP	
	r *	*p*
QTc on standard ECG recording	−0.1	NS
Premature ventricular beats (PVC)	0.1	NS
Sinusal QRS with QTc > 450 ms (no)	0.32	0.014
QTc (ms)	0.37	0.0035
Max QT (ms)	−0.05	NS
Max QTc (ms)	0.08	NS

* Pearson or Spearman correlation coefficient (as appropriate).

## Data Availability

Not applicable.
